# The genome sequence of a cranefly, *Tipula unca *(Wiedemann, 1817)

**DOI:** 10.12688/wellcomeopenres.20298.1

**Published:** 2023-12-05

**Authors:** Olga Sivell, Judy Webb, Ryan Mitchell, Duncan Sivell, Natural History Museum Genome Acquisition Lab, Darwin Tree of Life Barcoding collective, Wellcome Sanger Institute Tree of Life programme, Wellcome Sanger Institute Scientific Operations: DNA Pipelines collective, Tree of Life Core Informatics collective, Darwin Tree of Life Consortium

**Affiliations:** 1 Natural History Museum, London, England, UK; 2 Independent researcher, Sligo Town, Ireland

**Keywords:** Tipula unca, cranefly, genome sequence, chromosomal, Diptera

## Abstract

We present a genome assembly from an individual female *Tipula unca* (a cranefly; Arthropoda; Insecta; Diptera; Tipulidae). The genome sequence is 692.2 megabases in span. Most of the assembly is scaffolded into 4 chromosomal pseudomolecules. The mitochondrial genome has also been assembled and is 16.57 kilobases in length.

## Species taxonomy

Eukaryota; Metazoa; Eumetazoa; Bilateria; Protostomia; Ecdysozoa; Panarthropoda; Arthropoda; Mandibulata; Pancrustacea; Hexapoda; Insecta; Dicondylia; Pterygota; Neoptera; Endopterygota; Diptera; Nematocera; Tipulomorpha; Tipuloidea; Tipulidae; Tipulinae; *Tipula*; *Beringotipula*; *Tipula unca* (Wiedemann, 1817)(NCBI:txid2867263).

## Background

*Tipula unca* is a species of Tipulidae, one of the cranefly families, and is the only member of the subgenus *Beringotipula* to be found in Europe ( Stubbs, 2021). The species is orange-brown in colour, with a dark dorsal stripe on the abdomen, relatively long antennae with bristles at the base and relatively broad, short wings (length 15–21 mm) compared to other British *Tipula*. The wings have a mottled appearance. The male genitalia are distinctive with a dark tip to the abdomen that is bent upwards, with a yellow underside showing a double-U pattern ( Boardman, 2016; Stubbs, 2021).

There are four larval instars and the appearance of the first instar is considerably different to the remaining stages. The larvae live in soil and *T. unca* overwinters as a larva ( Stubbs, 2021). The pupa is exposed when the skin of the fourth instar larva is shed. This stage is short and lasts from a few days to approximately two weeks. The first instar larva was described by Hemmingsen (1965) and presented in detail by Podeniene *et al.* (2019) A description of the fourth instar larva and an identification key was provided by Chiswell (1956).

*Tipula unca* is common and widely distributed in Britain ( Stubbs, 1992). It prefers habitats with marshy ground, moist to saturated soil or under moss; it can be found in wet woods, by ponds and streams, but also in more open situations with marshy vegetation in cool districts ( Boardman, 2016; Dufour, 1986; Stubbs, 2021). The adults are on the wing from June to July ( Chiswell, 1956; Stubbs, 2021) and may be caught in light traps ( Dufour, 1986).

The eggs and first instar larvae of *Tipula* are particularly prone to desiccation. Large numbers of larvae and adults become prey of birds, especially starlings, and other vertebrates, such as bats, badgers, hedgehogs, shrews, moles, foxes ( Stubbs, 2021). The mortality of larvae caused by parasites is insignificant. In the study conducted by ( Carter, 1976), larvae of *Tipula unca* were found to be infected by the nuclear polyhedrosis virus (NPV), intestinal gregarines *Gregarina* spp., *Actinocephalus tipulae*, Coccidia and Thelastomatidae nematodes. *Gregarina* species were the most common parasites of *Tipula*, present in the intestines of most larvae when examined upon collection. Gregarines and Coccidia infect the gut of the larvae, usually causing no significant damage. The NPV was uncommon, but lethal. Many fourth instar larvae contained Thelastomatidae nematodes (up to 4 mm long) in the hindgut and occasionally in the mid-gut ( Carter, 1976). The rare and lethal infection with invertebrate iridoviruses (IIV) may result in the blue colouration of a specimen and has been reported for a pair of adult *Tipula cf. unca* by Boumans and Aarnes (2010).

The high-quality genome of *Tipula unca* was sequenced as part of the Darwin Tree of Life Project, a collaborative effort to sequence all named eukaryotic species in the Atlantic Archipelago of Britain and Ireland. Here we present a chromosomally complete genome sequence for *Tipula unca,* based on a female specimen (NHMUK014037116) from Cothill Fen National Nature Reserve.

## Genome sequence report

**Figure 1.**
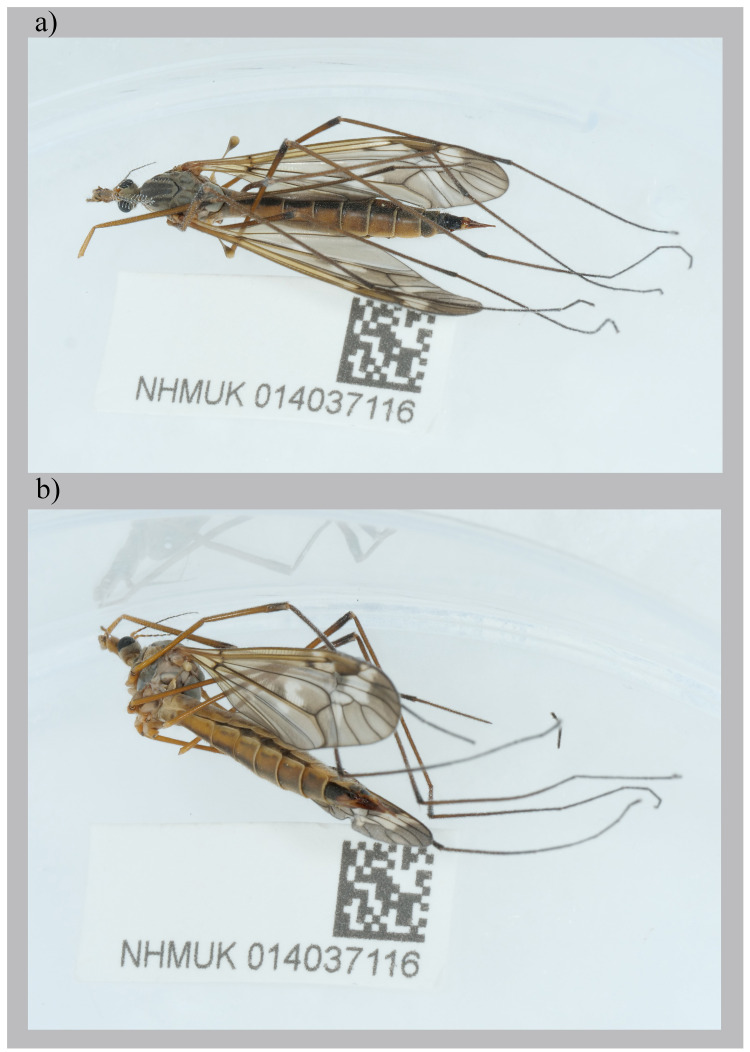
*Tipula unca* Wiedemann, 1817. The specimen (NHMUK014037116; idTipUnca1) used for genome sequencing: **a**) dorsal view, **b**) lateral view.

The genome was sequenced from one *Tipula unca* ( Figure 1) collected from Cothill Fen National Nature Reserve, EK (51.69, –1.33). A total of 39-fold coverage in Pacific Biosciences single-molecule HiFi long was generated. Primary assembly contigs were scaffolded with chromosome conformation Hi-C data. Manual assembly curation corrected 66 missing joins or mis-joins and removed 7 haplotypic duplications, reducing the assembly length by 0.62% and the scaffold number by 44.74%.

**Figure 2.**
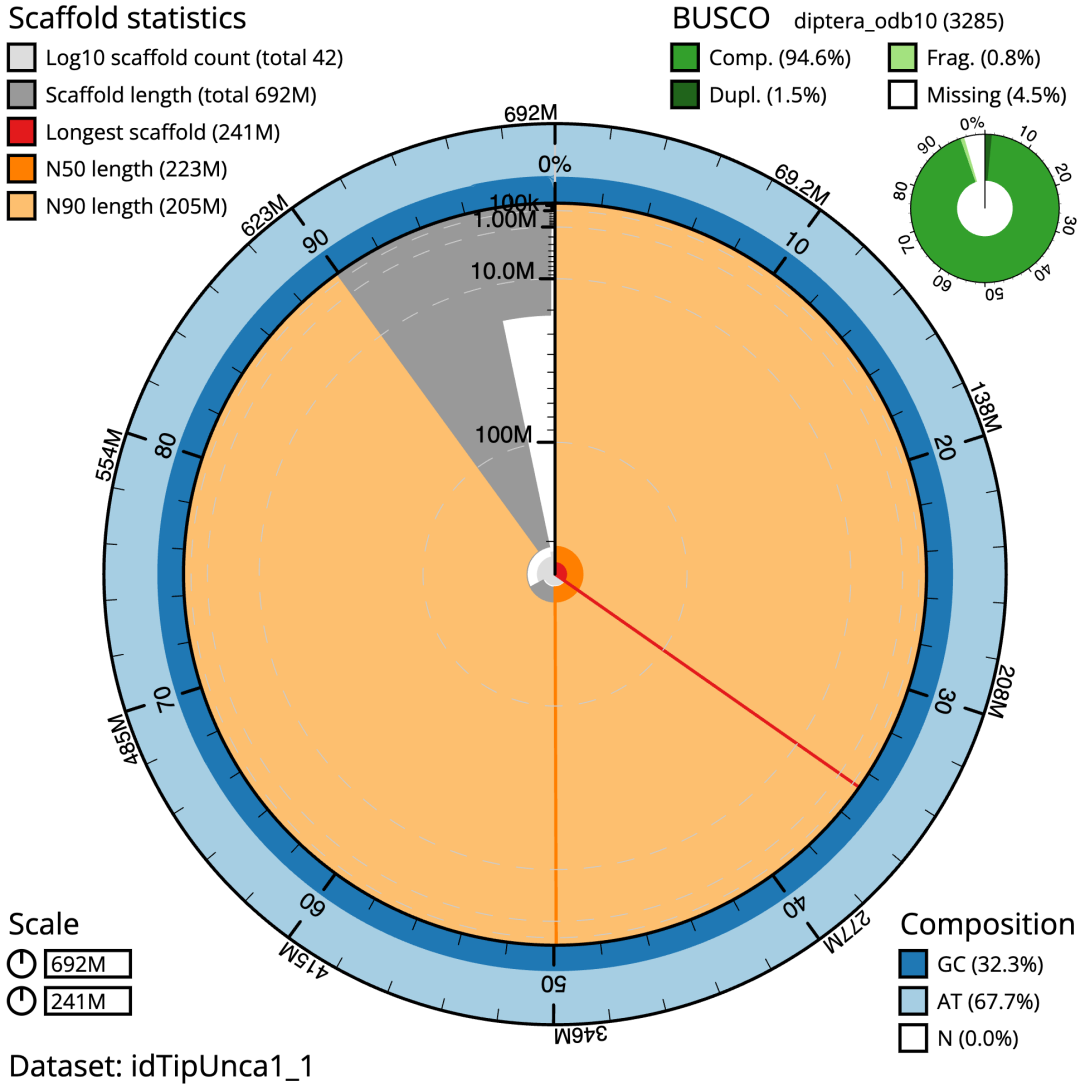
Genome assembly of *Tipula unca*, idTipUnca1.1: metrics. The BlobToolKit Snailplot shows N50 metrics and BUSCO gene completeness. The main plot is divided into 1,000 size-ordered bins around the circumference with each bin representing 0.1% of the 692,223,074 bp assembly. The distribution of sequence lengths is shown in dark grey with the plot radius scaled to the longest sequence present in the assembly (240,654,870 bp, shown in red). Orange and pale-orange arcs show the N50 and N90 sequence lengths (223,236,985 and 205,201,256 bp), respectively. The pale grey spiral shows the cumulative sequence count on a log scale with white scale lines showing successive orders of magnitude. The blue and pale-blue area around the outside of the plot shows the distribution of GC, AT and N percentages in the same bins as the inner plot. A summary of complete, fragmented, duplicated and missing BUSCO genes in the diptera_odb10 set is shown in the top right. An interactive version of this figure is available at https://blobtoolkit.genomehubs.org/view/Tipula%20unca/dataset/idTipUnca1_1/snail.

**Figure 3.**
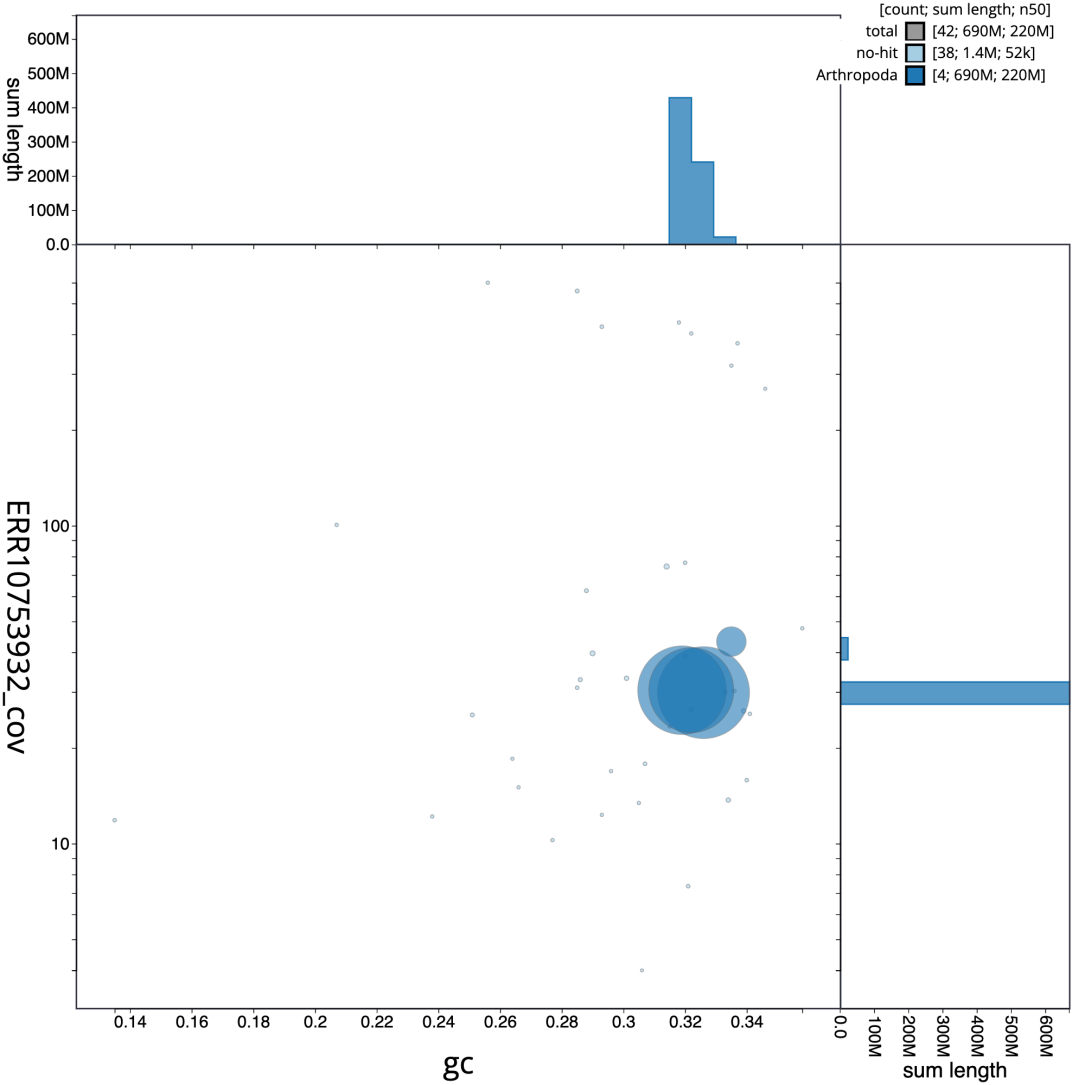
Genome assembly of *Tipula unca*, idTipUnca1.1: BlobToolKit GC-coverage plot. Scaffolds are coloured by phylum. Circles are sized in proportion to scaffold length. Histograms show the distribution of scaffold length sum along each axis. An interactive version of this figure is available at https://blobtoolkit.genomehubs.org/view/Tipula%20unca/dataset/idTipUnca1_1/blob.

**Figure 4.**
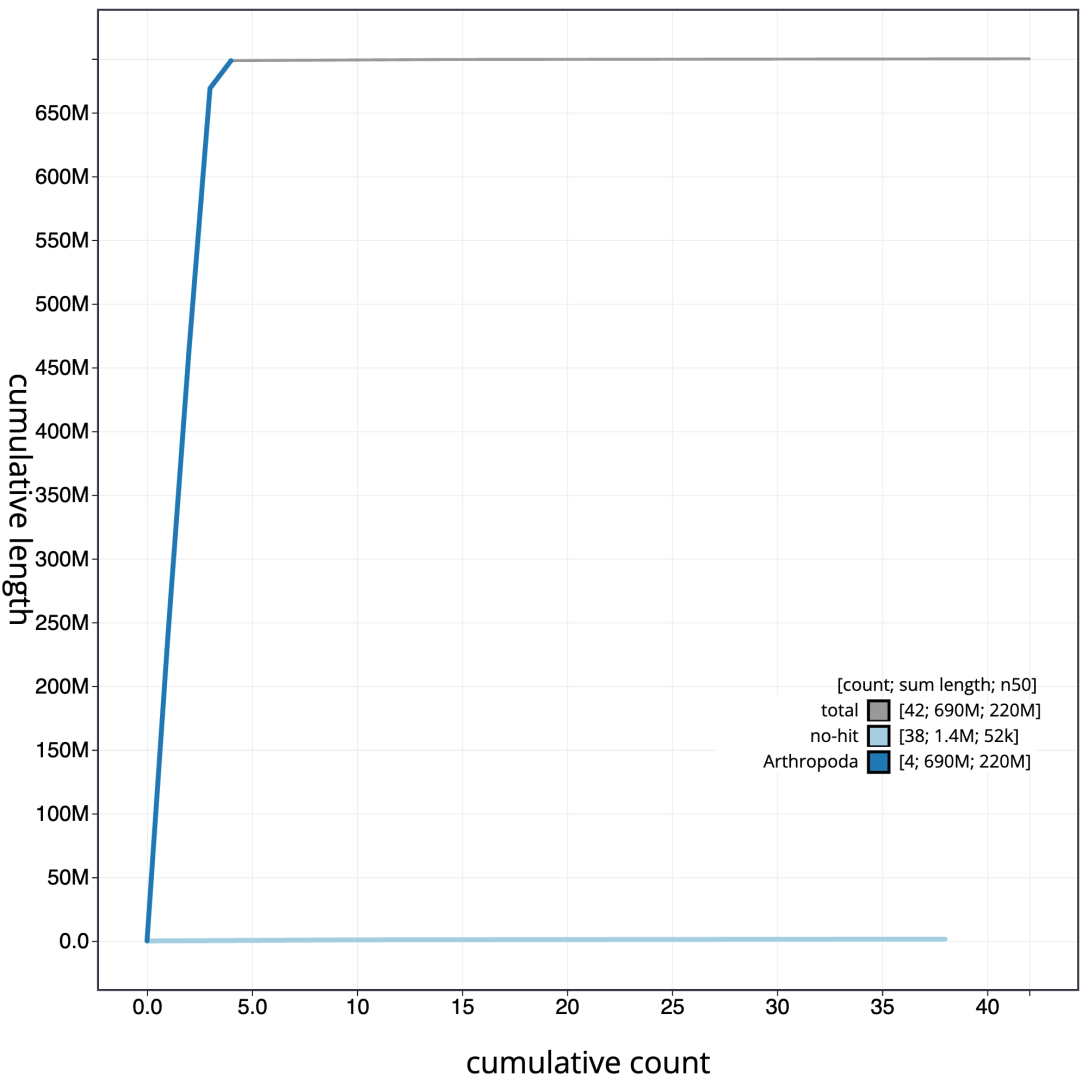
Genome assembly of *Tipula unca*, idTipUnca1.1: BlobToolKit cumulative sequence plot. The grey line shows cumulative length for all scaffolds. Coloured lines show cumulative lengths of scaffolds assigned to each phylum using the buscogenes taxrule. An interactive version of this figure is available at https://blobtoolkit.genomehubs.org/view/Tipula%20unca/dataset/idTipUnca1_1/cumulative.

**Figure 5.**
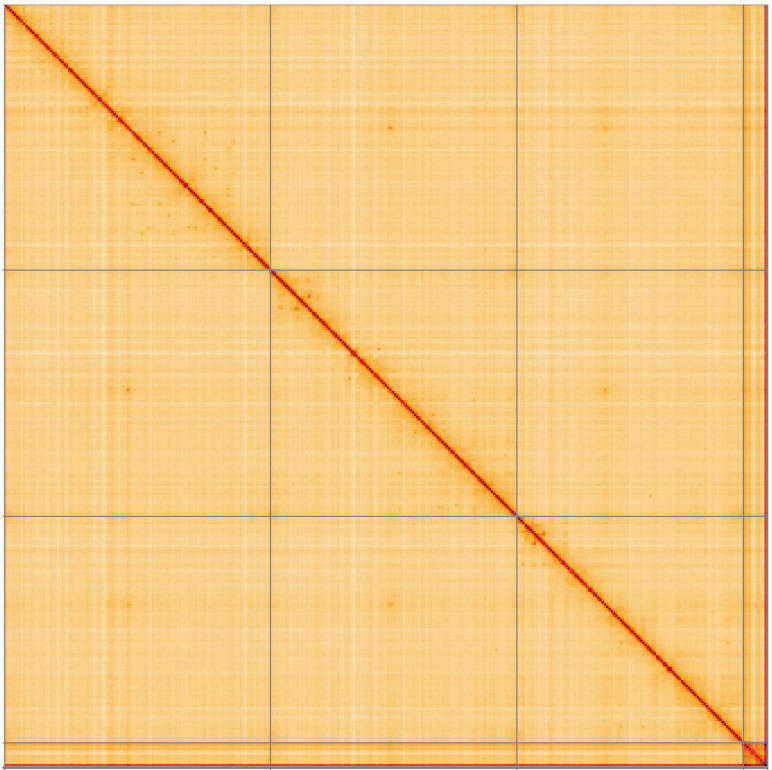
Genome assembly of *Tipula unca*, idTipUnca1.1: Hi-C contact map of the idTipUnca1.1 assembly, visualised using HiGlass. Chromosomes are shown in order of size from left to right and top to bottom. An interactive version of this figure may be viewed at https://genome-note-higlass.tol.sanger.ac.uk/l/?d=btxqqPz1Tbu1jDC6f4-X4A.

The final assembly has a total length of 692.2 Mb in 41 sequence scaffolds with a scaffold N50 of 223.2 Mb ( Table 1). The snailplot in Figure 2 provides a summary of the assembly statistics, while the distribution of assembly scaffolds on GC proportion and coverage is shown in Figure 3. The cumulative assembly plot in Figure 4 shows curves for subsets of scaffolds assigned to different phyla. Most (99.87%) of the assembly sequence was assigned to 4 chromosomal-level scaffolds. Chromosome-scale scaffolds confirmed by the Hi-C data are named in order of size ( Figure 5; Table 2). This specimen was assigned as female as the chromosomes were homogametic. We did not identify the sex chromosome, because sequence data from the heterogametic sex for *Tipula unca* was not available. Homology is unreliable for sex chromosome identification in Diptera due to frequent sex chromosome turnover ( Vicoso & Bachtrog, 2015). While not fully phased, the assembly deposited is of one haplotype. Contigs corresponding to the second haplotype have also been deposited. The mitochondrial genome was also assembled and can be found as a contig within the multifasta file of the genome submission.

**Table 1.** Genome data for *Tipula unca*, idTipUnca1.1.

Project accession data		
Assembly identifier	idTipUnca1.1	
Assembly release date	2023-06-16	
Species	*Tipula unca*	
Specimen	idTipUnca1	
NCBI taxonomy ID	2867263	
BioProject	PRJEB58671	
BioSample ID	SAMEA11025030	
Isolate information	idTipUnca1, female: abdomen (DNA sequencing); head and thorax (Hi-C data)	
Assembly metrics *		*Benchmark*
Consensus quality (QV)	58.4	*≥ 50*
*k*-mer completeness	100%	*≥ 95%*
BUSCO **	C:94.6%[S:93.2%,D:1.5%], F:0.8%,M:4.5%,n:3,285	*C ≥ 95%*
Percentage of assembly mapped to chromosomes	99.87%	*≥ 95%*
Sex chromosomes	Not identified	*localised homologous pairs*
Organelles	Mitochondrial genome assembled	*complete single alleles*
Raw data accessions		
PacificBiosciences SEQUEL II	ERR10753932	
Hi-C Illumina	ERR10742416	
Genome assembly		
Assembly accession	GCA_951394425.1	
*Accession of alternate * *haplotype*	GCA_951394375.1	
Span (Mb)	692.2	
Number of contigs	377	
Contig N50 length (Mb)	4.5	
Number of scaffolds	41	
Scaffold N50 length (Mb)	223.2	
Longest scaffold (Mb)	240.7	

* Assembly metric benchmarks are adapted from column VGP-2020 of “Table 1: Proposed standards and metrics for defining genome assembly quality” from Rhie *et al.* (2021). ** BUSCO scores based on the diptera_odb10 BUSCO set using v5.3.2. C = complete [S = single copy, D = duplicated], F = fragmented, M = missing, n = number of orthologues in comparison. A full set of BUSCO scores is available at https://blobtoolkit.genomehubs.org/view/Tipula%20unca/dataset/idTipUnca1_1/busco.

**Table 2.** Chromosomal pseudomolecules in the genome assembly of *Tipula unca*, idTipUnca1.

INSDC accession	Chromosome	Length (Mb)	GC%
OX596380.1	1	240.65	32.5
OX596381.1	2	223.24	32.0
OX596382.1	3	205.2	32.0
OX596383.1	4	21.7	33.5
OX596384.1	MT	0.02	25.5

The estimated Quality Value (QV) of the final assembly is 58.4 with *k*-mer completeness of 100%, and the assembly has a BUSCO v5.3.2 completeness of 94.6% (single = 93.2%, duplicated = 1.5%), using the diptera_odb10 reference set ( *n* = 3,285).

Metadata for specimens, barcode results, spectra estimates, sequencing runs, contaminants and pre-curation assembly statistics are given at https://links.tol.sanger.ac.uk/species/2867263.

## Methods

### Sample acquisition and nucleic acid extraction

A *Tipula unca* (specimen ID NHMUK014037116, ToLID idTipUnca1) was collected from Cothill Fen National Nature Reserve, England, UK (latitude 51.69, longitude –1.33) on 2021-06-19 using an aerial net. The specimen was collected by Judy Webb, Olga Sivell and Ryan Mitchell (Natural History Museum) and identified by Duncan Sivell (Natural History Museum) and preserved in liquid nitrogen.

The sample was prepared for DNA extraction at the Tree of Life laboratory, Wellcome Sanger Institute (WSI). The idTipUnca1 sample was weighed and dissected on dry ice with tissue set aside for Hi-C sequencing (as per the protocol https://dx.doi.org/10.17504/protocols.io.x54v9prmqg3e/v1). For sample homogenisation, tissue from the abdomen was disrupted using a Nippi Powermasher fitted with a BioMasher pestle ( https://dx.doi.org/10.17504/protocols.io.5qpvo3r19v4o/v1). DNA was extracted at the Wellcome Sanger Institute (WSI) Scientific Operations core using the Qiagen MagAttract HMW DNA kit, according to the manufacturer’s instructions.

All protocols developed by the Tree of Life laboratory are publicly available on protocols.io ( https://dx.doi.org/10.17504/protocols.io.8epv5xxy6g1b/v1).

### Sequencing

Pacific Biosciences HiFi circular consensus DNA sequencing libraries were constructed according to the manufacturers’ instructions. DNA sequencing was performed by the Scientific Operations core at the WSI on a Pacific Biosciences SEQUEL II (HiFi) instrument. Hi-C data were also generated from head and thorax tissue of idTipUnca1 using the Arima2 kit and sequenced on the Illumina NovaSeq 6000 instrument.

### Genome assembly, curation and evaluation

Assembly was carried out with Hifiasm ( Cheng *et al.*, 2021) and haplotypic duplication was identified and removed with purge_dups ( Guan *et al.*, 2020). The assembly was then scaffolded with Hi-C data ( Rao *et al.*, 2014) using YaHS ( Zhou *et al.*, 2023). The assembly was checked for contamination and corrected as described previously ( Howe *et al.*, 2021). Manual curation was performed using gEVAL, HiGlass ( Kerpedjiev *et al.*, 2018) and Pretext ( Harry, 2022). The mitochondrial genome was assembled using MitoHiFi ( Uliano-Silva *et al.*, 2023), which runs MitoFinder ( Allio *et al.*, 2020) or MITOS ( Bernt *et al.*, 2013) and uses these annotations to select the final mitochondrial contig and to ensure the general quality of the sequence.

A Hi-C map for the final assembly was produced using bwa-mem2 ( Vasimuddin *et al.*, 2019) in the Cooler file format ( Abdennur & Mirny, 2020). To assess the assembly metrics, the *k*-mer completeness and QV consensus quality values were calculated in Merqury ( Rhie *et al.*, 2020). This work was done using Nextflow ( Di Tommaso *et al.*, 2017) DSL2 pipelines “sanger-tol/readmapping” ( Surana *et al.*, 2023a) and “sanger-tol/genomenote” ( Surana *et al.*, 2023b). The genome was analysed within the BlobToolKit environment ( Challis *et al.*, 2020) and BUSCO scores ( Manni *et al.*, 2021; Simão *et al.*, 2015) were calculated.

Table 3 contains a list of relevant software tool versions and sources.

**Table 3.** Software tools: versions and sources.

Software tool	Version	Source
BlobToolKit	4.2.1	https://github.com/blobtoolkit/blobtoolkit
BUSCO	5.3.2	https://gitlab.com/ezlab/busco
Hifiasm	0.16.1-r375	https://github.com/chhylp123/hifiasm
HiGlass	1.11.6	https://github.com/higlass/higlass
Merqury	MerquryFK	https://github.com/thegenemyers/MERQURY.FK
MitoHiFi	2	https://github.com/marcelauliano/MitoHiFi
PretextView	0.2	https://github.com/wtsi-hpag/PretextView
purge_dups	1.2.3	https://github.com/dfguan/purge_dups
sanger-tol/genomenote	v1.0	https://github.com/sanger-tol/genomenote
sanger-tol/ readmapping	1.1.0	https://github.com/sanger-tol/readmapping/tree/1.1.0
YaHS	1.2a	https://github.com/c-zhou/yahs

### Wellcome Sanger Institute – Legal and Governance

The materials that have contributed to this genome note have been supplied by a Darwin Tree of Life Partner. The submission of materials by a Darwin Tree of Life Partner is subject to the **‘Darwin Tree of Life Project Sampling Code of Practice’**, which can be found in full on the Darwin Tree of Life website here. By agreeing with and signing up to the Sampling Code of Practice, the Darwin Tree of Life Partner agrees they will meet the legal and ethical requirements and standards set out within this document in respect of all samples acquired for, and supplied to, the Darwin Tree of Life Project.

Further, the Wellcome Sanger Institute employs a process whereby due diligence is carried out proportionate to the nature of the materials themselves, and the circumstances under which they have been/are to be collected and provided for use. The purpose of this is to address and mitigate any potential legal and/or ethical implications of receipt and use of the materials as part of the research project, and to ensure that in doing so we align with best practice wherever possible. The overarching areas of consideration are:

•     Ethical review of provenance and sourcing of the material

•     Legality of collection, transfer and use (national and international)

Each transfer of samples is further undertaken according to a Research Collaboration Agreement or Material Transfer Agreement entered into by the Darwin Tree of Life Partner, Genome Research Limited (operating as the Wellcome Sanger Institute), and in some circumstances other Darwin Tree of Life collaborators.

## Data Availability

European Nucleotide Archive: *Tipula unca*. Accession number PRJEB58671; https://identifiers.org/ena.embl/PRJEB58671 ( Wellcome Sanger Institute, 2023). The genome sequence is released openly for reuse. The *Tipula unca* genome sequencing initiative is part of the Darwin Tree of Life (DToL) project. All raw sequence data and the assembly have been deposited in INSDC databases. The genome will be annotated using available RNA-Seq data and presented through the Ensembl pipeline at the European Bioinformatics Institute. Raw data and assembly accession identifiers are reported in Table 1.

## Author information

Members of the Natural History Museum Genome Acquisition Lab are listed here: https://doi.org/10.5281/zenodo.7139035.

Members of the Darwin Tree of Life Barcoding collective are listed here: https://doi.org/10.5281/zenodo.4893703.

Members of the Wellcome Sanger Institute Tree of Life programme are listed here: https://doi.org/10.5281/zenodo.4783585.

Members of Wellcome Sanger Institute Scientific Operations: DNA Pipelines collective are listed here: https://doi.org/10.5281/zenodo.4790455.

Members of the Tree of Life Core Informatics collective are listed here: https://doi.org/10.5281/zenodo.5013541.

Members of the Darwin Tree of Life Consortium are listed here: https://doi.org/10.5281/zenodo.4783558.
