# Supplementary material for: The genome sequence of a cranefly, Tipula unca (Wiedemann, 1817)
Source: Wellcome Open Res. 2023 Dec 5;8:559. [Version 1] doi: 10.12688/wellcomeopenres.20298.1 (PMC11494271; doi:10.12688/wellcomeopenres.20298.1)
Supplement: Peer review history

# Reviewer report: Reviewer response for version 1

**DOI:** 10.21956/wellcomeopenres.22480.r106046

**Published:** 2024-10-21

**Authors:** Hans-Peter Fuehrer

**Affiliations:** 1 University of Veterinary Medicine, Vienna, Austria

The authors describe the genome sequence of one species of craneflies – *Tipula unca.*

 Morphology and biology of this species is described well.

 Please provide information in Section Background where Cothill Fen National Nature Reserve is located. It is given in Methods (However, for people outside the UK it is still unclear where in England it is located)

Are sufficient details of methods and materials provided to allow replication by others?

Yes

Is the rationale for creating the dataset(s) clearly described?

Yes

Are the datasets clearly presented in a useable and accessible format?

Yes

Are the protocols appropriate and is the work technically sound?

Yes

Reviewer Expertise:

Medical Entomology

I confirm that I have read this submission and believe that I have an appropriate level of expertise to confirm that it is of an acceptable scientific standard.

# Reviewer report: Reviewer response for version 1

**DOI:** 10.21956/wellcomeopenres.22480.r101955

**Published:** 2024-10-18

**Authors:** Fernando Díaz

**Affiliations:** 1 Colgate University, Hamilton, New York, USA

This paper describes the sequencing and assembly of the genome from a female cranefly, *Tipula unca*. The background provides some descriptive information about the species morphology, and the genome sequence report summarizes the sequencing data, assembly, curation and assembly quality. The paper uses robust methods leading to a chromosome-level genome. I only have a few suggestions:

 1. The background provides important information about the biology of the species. However, I believe it is missing some of the broader impacts of the data generated. Could the availability of a reference genome in this species help in understanding the phylogeny of the entire group? Similarly, are there important ecological and evolutionary questions in this species that can now be addressed with this genome? Some examples of current papers or questions will be helpful.

 2. The genome sequencing report mentions that a 39-fold coverage was generated in Pacific Biosciences single-molecule HiFi long reads (the word “reads” was missing). Then, contigs were scaffolded using chromosome conformation Hi-C data. A more detailed description or an initial table with the number of reads and coverage generated with these technologies will be beneficial for the reader. In addition, a better description of how one type of data informs the other for scaffolding will be appreciated by the reader. A brief clarification in the text about any contigs not fully ordered/oriented will also be beneficial.

 3. The benchmark of the final assembly quality is included In Table 1 (QV, k-mer, and BUSCO completeness). However, some readers would appreciate a brief description with the meaning of these estimates.

Are sufficient details of methods and materials provided to allow replication by others?

Yes

Is the rationale for creating the dataset(s) clearly described?

Partly

Are the datasets clearly presented in a useable and accessible format?

Partly

Are the protocols appropriate and is the work technically sound?

Yes

Reviewer Expertise:

Evolutionary Genomics.

I confirm that I have read this submission and believe that I have an appropriate level of expertise to confirm that it is of an acceptable scientific standard, however I have significant reservations, as outlined above.

# Reviewer report: Reviewer response for version 1

**DOI:** 10.21956/wellcomeopenres.22480.r77278

**Published:** 2024-05-20

**Authors:** Vinaya Shetty

**Affiliations:** 1 Department of Entomology, Texas A&M University, Texas, USA

Background:

1. Parasitism and Predation:
  - The description of parasites and predators of *Tipula unca *larvae provides valuable insight into the ecological pressures faced by the species. However, it would be useful to know more about the potential impact of these parasites and predators on the population dynamics of *Tipula unca* and their broader ecological implications.
  - Clarification on the rarity and lethality of certain infections, such as invertebrate iridoviruses (IIV), would help readers understand their significance in the context of cranefly biology.

Genome sequence report

 The Genome sequence report section offers a detailed account of the genome sequencing, assembly, curation, and evaluation processes for *Tipula unca*. While it provides valuable information, there are areas where additional clarification and detail could enhance the reader's understanding:

1. Sequencing and Assembly:
  - Further Details on the manual assembly curation process, such as the criteria used for identifying missing joins or mis-joins and haplotypic duplications, would provide transparency and insight into the quality control measures implemented.
2. Quality Assessment:
  - The section reports an estimated Quality Value (QV) of the final assembly, k-mer completeness, and BUSCO completeness. While these metrics provide insight into the quality and completeness of the assembly, it would be useful to briefly explain how these values were calculated and their significance in assessing genome quality. 
  - Sequencing and Assembly:
    - The section mentions the generation of 39-fold coverage in Pacific Biosciences HiFi long reads and the use of chromosome conformation Hi-C data for scaffolding. It would be beneficial to explain how these two sequencing technologies complement each other and contribute to the accuracy and completeness of the assembly.
    - Further details on the manual assembly curation process, such as the criteria used for identifying missing joins or mis-joins and haplotypic duplications, would provide transparency and insight into the quality control measures implemented.
  - Assembly Statistics:
    - The assembly statistics, including the total length, scaffold N50, and reduction in assembly length and scaffold number after curation, are provided. However, it would be helpful to explain the significance of these metrics in assessing the quality and contiguity of the assembly.

Methods:

- Additional information on the sequencing depth and coverage achieved with both sequencing platforms would give readers insight into the robustness of the generated data.
- More details on the parameters and settings used for genome assembly and curation would help readers understand the reproducibility and reliability of the results.
- While the section briefly mentions the evaluation of the assembly, it could benefit from further elaboration on the metrics used to assess the quality of the assembly and the criteria for determining completeness and accuracy.
- The inclusion of any manual curation steps or quality control measures implemented during the assembly process would provide transparency and insight into the rigor of the genomic analysis.

The Genome sequence report section presents detailed assembly statistics and quality assessment metrics. However, providing interpretation of these results and discussing their implications for understanding *Tipula unca* biology and evolution would enrich the manuscript.

Are sufficient details of methods and materials provided to allow replication by others?

Partly

Is the rationale for creating the dataset(s) clearly described?

Partly

Are the datasets clearly presented in a useable and accessible format?

Yes

Are the protocols appropriate and is the work technically sound?

Yes

Reviewer Expertise:

Genetics and molecular biology

I confirm that I have read this submission and believe that I have an appropriate level of expertise to confirm that it is of an acceptable scientific standard, however I have significant reservations, as outlined above.
